# Diffuse Synovial Lipomatosis and Meniscal Hypoplasia in an Adolescent Patient: A Case Report

**DOI:** 10.7759/cureus.110630

**Published:** 2026-06-10

**Authors:** Klaus Mieth, Jorge Rojas, German Rojas, María Andrea Montiel, María José Montoya Niño

**Affiliations:** 1 Department of Orthopedics and Traumatology, Hospital Universitario Fundación Santa Fe de Bogotá, Bogotá, COL; 2 Department of Orthopedics and Traumatology, School of Medicine, Universidad de Los Andes, Bogotá, COL

**Keywords:** adolescent, hypoplasia, knee, lipomatosis, meniscal

## Abstract

We present the case of a 16-year-old male with chronic left knee pain, progressive swelling, and limited mobility. Magnetic resonance imaging (MRI) revealed a combination of diffuse synovial lipomatosis and hypoplasia of both the medial and lateral menisci in the left knee. The patient underwent knee arthroscopy with synovectomy and biopsy, confirming the diagnosis of synovial lipomatosis. This case highlights the importance of recognizing the coexistence of congenital and proliferative synovial diseases and contributes to the literature on the diagnosis and management of these rare conditions.

## Introduction

Synovial lipomatosis, also known as lipoma arborescens, is a rare, benign condition characterized by the proliferation of adipose tissue within the synovial membrane of joints. It most commonly affects the knee but has also been reported in other joints, including the wrist, shoulder, and foot [1,2]. Clinical manifestations vary, with patients presenting with joint swelling, mechanical symptoms, and effusion, often leading to functional impairment. Given its non-specific presentation, synovial lipomatosis is frequently misdiagnosed as tenosynovial giant cell tumor (TGCT) or septic arthritis, necessitating an accurate differential diagnosis [3].

The precise etiology of synovial lipomatosis remains uncertain. However, associations with chronic inflammation, degenerative joint disorders, and prior trauma have been proposed. Some studies suggest that excessive intra-articular fat deposition, possibly influenced by metabolic or inflammatory processes, contributes to its development [3].

Histopathologically, it is characterized by villous proliferation of the synovium with infiltration of mature adipocytes and chronic inflammatory cells, including lymphocytes and plasma cells [4,5]. MRI is the imaging modality of choice, demonstrating synovial hypertrophy with high fat-signal intensity and effusion [5].

Meniscal hypoplasia is a rare congenital anomaly characterized by an underdeveloped meniscus. While other meniscal abnormalities such as discoid, double-layered, and ring-shaped menisci have been described, isolated meniscal hypoplasia remains poorly documented. The condition presents diagnostic challenges due to its variable clinical course, with some patients being asymptomatic and others developing joint instability or early degenerative changes [6,7].

The coexistence of meniscal hypoplasia with synovial lipomatosis is exceptionally rare, making this case valuable in expanding the current understanding of these conditions.

## Case presentation

A 16-year-old male from a rural area, with no significant past medical history, presented with a three-year history of progressive left knee swelling, pain, and restricted mobility. The patient reported an insidious onset of symptoms without a preceding traumatic event. Over time, he developed a fixed flexion deformity, which impaired his gait and limited his ability to participate in sports. He had previously received nonsteroidal anti-inflammatory drugs and physical therapy, but his symptoms persisted.

On physical examination, the patient exhibited an antalgic gait with a noticeable limp due to a fixed flexion contracture of 15 degrees and anterior knee impingement. There was diffuse synovial thickening, particularly in the suprapatellar pouch and medial gutter, accompanied by moderate effusion. The range of motion was restricted from 15 to 100 degrees, resulting in an effective arc of motion of 85 degrees. There were no signs of ligamentous instability. Neurological and vascular examinations were unremarkable, and there were no cutaneous manifestations suggestive of a systemic inflammatory disorder. Laboratory tests, including complete blood count, C-reactive protein, and rheumatoid factor, were within normal limits.

Radiographic evaluation included anteroposterior and lateral views of both knees. The images revealed residual open physes, soft tissue prominence, and an increased anteroposterior diameter of the left femoral condyle (60 mm) compared to the right (57 mm) (Figure 1). MRI demonstrated a septated lipomatous mass involving Hoffa's fat pad, the suprapatellar recess, and the anteromedial compartment. Additionally, a morphological alteration in meniscal size was observed macroscopically. Measurements obtained on coronal views demonstrated a thickness of 2 mm for the internal meniscus and 5 mm for the external meniscus. On sagittal views, the external meniscus measured 6.6 mm in both the anterior and posterior horns, while the internal meniscus measured 6 mm anteriorly and 7.9 mm posteriorly (Figure 2).

**Figure 1:**
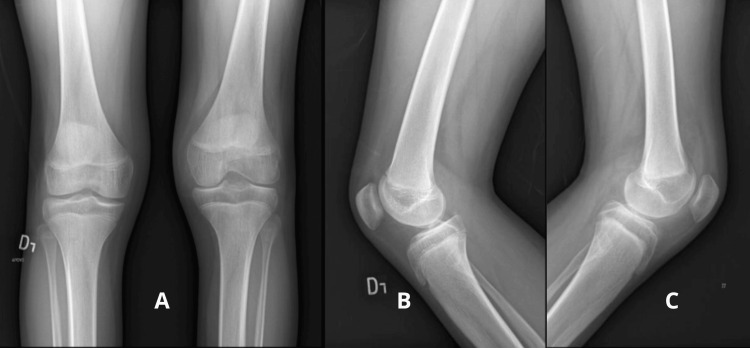
Preoperative Radiographs of the Knees (A) Anteroposterior (AP) view of both knees demonstrating open physes, soft tissue prominence, and preserved joint spaces in the left knee (B) Lateral view of the right knee showing an anteroposterior femoral condyle diameter of 57 mm (C) Lateral view of the left knee showing an anteroposterior femoral condyle diameter of 60 mm, with a mild increase in soft tissue volume.

**Figure 2:**
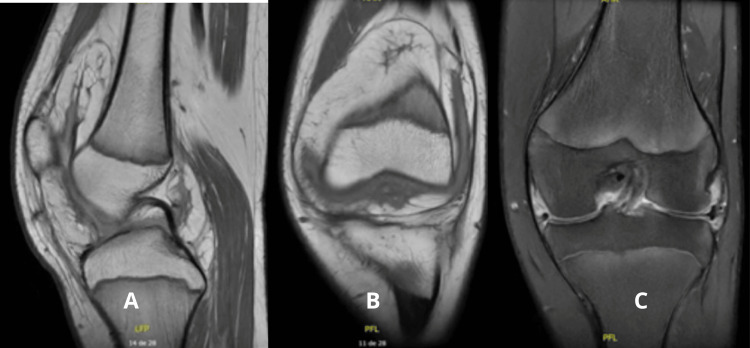
Magnetic Resonance Imaging (MRI) of the Left Knee (A) Sagittal and coronal T1-weighted MRI images showing increased fluid in the subsynovial region of the subquadricipital area and the anteromedial compartment. A subsynovial lipomatous mass is evident within the subquadricipital space, the anteromedial compartment, and the Hoffa’s fat pad. (B-C) Coronal T2-weighted MRI image demonstrating hypoplasia of both the medial and lateral menisci.

Based on clinical and paraclinical findings, an intra-articular mass of the left knee was identified, with a differential diagnosis including synovial lipomatosis versus idiopathic synovial hyperplasia. The patient presented with a severe flexion deformity and anterior impingement, resulting in limitations in activities of daily living due to pain and deformity, as well as complete restriction of sports participation. Given these findings, a left knee arthroscopy was indicated with the following objectives: diagnostic evaluation, tissue biopsy, and therapeutic orientation, aiming for complete resection of the pathological tissue to restore knee function and resolve anterior impingement.

The patient underwent arthroscopic synovectomy using standard portals, including medial and lateral parapatellar portals, a lateral suprapatellar portal, and two accessory portals (posteromedial and posterolateral). Intraoperatively, a diffuse villous proliferation of yellowish adipose tissue and a macroscopically reduced size of the meniscus was observed, confirming the MRI findings (Figures 2, 3). Systematic resection of the pathological synovium was performed, and multiple biopsy specimens were obtained. Histopathological examination revealed chronic synovitis with villous proliferation and mature adipose tissue infiltration, consistent with synovial lipomatosis. 

**Figure 3:**
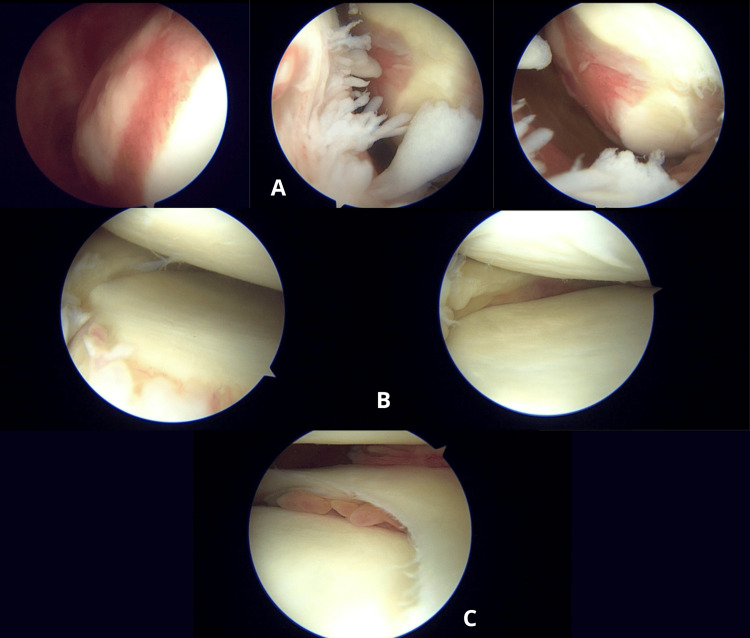
Arthroscopic Assessment of the Left Knee: Intraoperative Images (A) Pannus and synovial hyperplasia in the trochlea and internal condyle, (B) Hypoplasia of the internal meniscus, (C) Hypoplasia of the external meniscus with hyperplasic synovial

Postoperative course

The patient was instructed to ambulate with two crutches, with progressive weight-bearing as tolerated. Physical therapy focused on achieving full knee extension and improving flexion through active-assisted exercises performed in both supine and prone positions. Additional modalities included diathermy and ultrasound for pain control, along with quadriceps re-education. Once edema was controlled, isometric quadriceps exercises and pelvic girdle strengthening were initiated. No orthoses or braces were prescribed. The patient used crutches for four weeks.

Medication consisted of acetaminophen for two weeks. No additional measures were implemented to prevent arthrofibrosis in the immediate postoperative period.

At one month postoperatively, the patient exhibited a stable gait. Mild knee effusion persisted, but the surgical incisions were well-healed. The active range of motion had improved to 0-100 degrees. Once full knee extension was achieved, physical examination revealed a suspected limb length discrepancy of the left lower extremity. A standing full-length radiograph was obtained, demonstrating a 13 mm length discrepancy of the left lower limb, consisting of 10 mm at the femoral level and 3 mm at the tibial level. 

From the fourth postoperative week, conditioning exercises including stationary cycling and treadmill walking were introduced. By the second postoperative month, the patient reported no limping, with further improvement in the range of motion to 0-125 degrees. Inflammatory markers remained within normal limits, and sports restrictions were maintained. A gradual return to sports activity was allowed after the second postoperative month.

At five months, he achieved full pain-free ambulation with near-complete restoration of knee motion (0-128 degrees) and was cleared for light to moderate physical activity. Despite the favorable recovery, he did not attend scheduled rheumatology follow-up, preventing a comprehensive evaluation for an underlying inflammatory disorder.

## Discussion

This case presents a rare coexistence of synovial lipomatosis and hypoplasia of both the internal and external menisci in the left knee, a combination that has been described only once before. A prior report by Utkan et al. documented a 37-year-old patient with both conditions, but our case differs in age of onset, diffuse intra-articular involvement, and absence of predisposing inflammatory or degenerative conditions [8]. This makes our case particularly relevant for expanding the understanding of pediatric presentations of these conditions and their potential interrelation.

Synovial lipomatosis is a benign proliferative condition of the synovial membrane, predominantly affecting the knee. It is characterized by abnormal adipose deposition within the synovium, leading to joint swelling, mechanical symptoms, and effusion [1,2]. While commonly described in middle-aged adults, cases in children and adolescents have been documented, including a 15-year-old girl with chronic knee pain treated with arthroscopic excision [7], an eight-year-old girl with concurrent chondroblastoma [9], and an adolescent initially misdiagnosed with juvenile idiopathic arthritis before MRI confirmed synovial lipomatosis [5]. The adolescent onset observed in this case is rare, suggesting that alternative pathogenic mechanisms such as congenital factors or early metabolic alterations may play a role.

Furthermore, most reported cases of synovial lipomatosis are localized to the suprapatellar pouch. In contrast, our patient exhibited a widespread lipomatous mass affecting Hoffa’s fat pad, the suprapatellar region, the anteromedial compartment, making this an atypical distribution of disease. The classification by Soler et al. describes three patterns based on MRI findings, yet it is unclear whether diffuse cases such as this one fit within this system [10].

MRI remains the gold standard for diagnosis, revealing characteristic villous proliferations with high fat signal intensity [5]. In this patient, MRI findings of septated lipomatous masses throughout the joint led to histopathological evaluation, which confirmed villous proliferation of the synovium with mature adipose infiltration, consistent with prior descriptions of synovial lipomatosis [4].

The hypoplasia of both the internal and external menisci in the left knee is another remarkable aspect of this case. Meniscal hypoplasia is a rare congenital anomaly that results in reduced meniscal volume, leading to altered biomechanics and a potential risk of early joint degeneration [6,7]. To our knowledge, no formal diagnostic criteria for meniscal hypoplasia have been established in the pediatric orthopedic literature. However, we defined meniscal hypoplasia as the presence of two or three meniscus MRI measurement parameters below the age-group mean reported by Zhu et al. [11], with no history of prior surgery, trauma, or systemic disease. In our patient, MRI demonstrated width and thickness of the anterior horn, posterior horn and body of the internal and external meniscus, all below the age-group reference mean for age group D (15 to 18 years) (Tables 1, 2). These findings, in the absence of any confounding pathology, support the diagnosis of meniscal hypoplasia.

**Table 1:** External Meniscal MRI Measurements: Normative Reference Values by Age Group, Zhu et al. and Patient Measurements All measurements in millimeters (mm). Values presented as mean ± standard deviation. Normative data source: Zhu T et al. [11]. Morphological data of the external meniscus. Unit: mm. EMAT: external meniscal anterior horn thickness. EMAW: External meniscal anterior horn width. EMBT: External meniscal body thickness. EMBW: External meniscal body width. EMPT: External meniscal posterior horn thickness. EMPW: external meniscal posterior horn width.

	External meniscus anterior horn		External meniscus body		External meniscus posterior horn	
	EMAW	EMAT	EMBW	EMBT	EMPW	EMPT
15-18 Years Old	8.60±1.56	4.39±0.70	10.09±1.95	7.12±1.19	9.47±1.15	6.26±0.96
Patient Measurement	6.6	1.8	6.2	4.8	6.6	3.6

**Table 2:** Internal Meniscal MRI Measurements: Normative Reference Values by Age Group, Zhu et al. and Patient Measurements All measurements in millimeters (mm). Values presented as mean ± standard deviation. Normative data source: Zhu T et al. [11]. Morphological data of the internal meniscus. Unit: mm. IMAT: internal meniscal anterior horn thickness. IMAW: internal meniscal anterior horn width. IMBT: internal meniscal body thickness. IMBW: internal meniscal body width. IMPT: internal meniscal posterior horn thickness. IMPW: internal meniscal posterior horn width.

	Internal meniscus anterior horn		Internal meniscus body		Internal meniscus posterior horn	
	IMAW	IMAT	IMBW	IMBT	IMPW	IMPT
15-18 Years Old	6.98±1.10	5.42±1.04	8.06±1.18	6.29±0.96	11.49±0.83	5.53±0.95
Patient Measurement	6.1	2.3	3.2	1.8	7.9	1.9

The biomechanical implications of meniscal hypoplasia in a skeletally immature patient warrant attention. The meniscus transmits 50-70% of the axial tibiofemoral load and is essential for contact stress distribution and cartilage protection [12]. Reduced tibial plateau coverage secondary to an undersized meniscus has been independently associated with cartilage loss and osteoarthritis progression [13,14], a risk that is particularly consequential in the pediatric population, where meniscal compromise has been linked to radiographic osteoarthritis in up to 40% of adolescent patients over time [15]. In our patient, the reduced sagittal diameter and body width imply a smaller effective contact area and elevated peak cartilage pressures during weight-bearing, analogous to the biomechanical profile seen after partial meniscectomy [16]. Long-term surveillance for early degenerative changes is therefore warranted.

Unlike previous reports where meniscal hypoplasia was associated with instability or trauma, our patient did not exhibit ligamentous laxity, mechanical symptoms, or meniscal tears, suggesting a more stable variant of the condition.

Since the contralateral knee was not evaluated, we cannot determine whether this represents a unilateral or bilateral developmental abnormality. While the coexistence of synovial lipomatosis and meniscal hypoplasia is unusual, any potential pathophysiological relationship remains speculative. Given the extreme rarity of both conditions, it is unlikely that a clear association will ever be established. Their coexistence in this case may be incidental, though further documentation could help explore possible shared developmental or biomechanical factors. Further studies should explore whether meniscal hypoplasia in pediatric patients may predispose them to synovial lipomatosis, as chronic instability or altered synovial biomechanics could contribute to excessive adipose infiltration into the synovial membrane.

Another unique finding was the increased anteroposterior diameter of the left femoral condyle (60 mm vs. 57 mm on the contralateral side). While bilateral asymmetry of articular surface dimensions has been documented as a normal adaptive response to differential mechanical loading during skeletal development, and normative studies report considerable interindividual variability in femoral AP dimensions across populations (range 54-66 mm) [17], a 3 mm unilateral increase in an otherwise skeletally immature adolescent may exceed expected physiological asymmetry. Chronic synovitis is known to alter periarticular bone biology through increased intra-articular pressure, synovial hyperemia, and osteoclast-mediated remodeling, and untreated lipoma arborescens has been associated with progressive structural joint changes when synovectomy is delayed [18]. We therefore propose that the observed condylar enlargement may represent adaptive remodeling secondary to longstanding synovial hypertrophy and effusion, rather than pure anatomical variation. There are no prior reports directly correlating synovial lipomatosis with focal femoral condyle hypertrophy, making this an intriguing radiological finding that warrants further investigation.

The limb length discrepancy of 13 mm, with the left leg longer than the right, also lacks a definitive explanation. Possible causes include asymmetric femoral or tibial growth due to chronic synovial hyperplasia and inflammation or an unrelated developmental variation. Given the lack of data on limb overgrowth in synovial lipomatosis, future studies should evaluate whether chronic synovial disease in childhood or adolescence could contribute to skeletal asymmetries.

The management of synovial lipomatosis is primarily arthroscopic synovectomy, which serves both therapeutic and diagnostic purposes. In the largest available study, Wang et al. reviewed 71 cases treated with arthroscopic resection, reporting a recurrence rate of only 2.8% with a mean follow-up of 15 months [19]. Our patient showed significant improvement following synovectomy, with resolution of swelling, pain relief, and improved range of motion at five months, supporting the efficacy of synovectomy in younger patients, though long-term monitoring is necessary [20]. In contrast, meniscal hypoplasia lacks a defined treatment strategy. Given that our patient did not exhibit instability, locking, or symptoms directly attributed to the meniscal deficiency, a conservative approach was chosen. While literature on meniscal hypoplasia and its long-term consequences is limited, previous reports suggest that meniscal deficiencies can lead to altered knee biomechanics, potentially predisposing patients to early-onset osteoarthritis. Surveillance is essential to monitor for potential degenerative changes over time.

This case highlights the importance of considering congenital and proliferative synovial conditions in young patients presenting with chronic knee pain and restricted motion. Key strengths of this report are the detailed imaging analysis and histopathological confirmation, which strengthen the diagnostic accuracy and exclude alternative diagnoses such as pigmented villonodular synovitis or chronic inflammatory arthritis. Additionally, documenting this case in an adolescent patient contributes valuable insight to the limited literature on pediatric synovial lipomatosis and meniscal hypoplasia, particularly regarding their potential coexistence and long-term implications. However, certain limitations must be acknowledged. The contralateral knee was not evaluated, which prevents a definitive determination of whether the meniscal hypoplasia was a unilateral anomaly or part of a broader developmental abnormality. Furthermore, the lack of long-term follow-up makes it difficult to assess the risk of disease progression, recurrence of synovial lipomatosis, or potential degenerative changes associated with meniscal hypoplasia. Future studies should aim to clarify whether early surgical intervention in meniscal hypoplasia could modify long-term joint health outcomes.

## Conclusions

This case expands the understanding of synovial lipomatosis and the coexistence with hypoplasia of both the internal and external menisci in a single knee as a distinct but potentially related intra-articular condition, yet scarcely documented in the literature. The early-onset presentation and diffuse intra-articular involvement distinguish it from typical cases of synovial lipomatosis and raise important considerations for diagnosis, classification, and long-term management. In addition, MRI plays a critical role in diagnosis, revealing extensive disease beyond existing classification patterns. Arthroscopic synovectomy remains the treatment of choice for symptomatic synovial lipomatosis, though long-term surveillance is necessary, while meniscal hypoplasia requires careful surveillance to prevent degenerative changes. Given the rarity of this association, further research and case documentation are needed to explore potential shared mechanisms, long-term clinical course and outcomes of this rare coexistence and improve clinical management strategies.

## References

[1] Siva C, Brasington R, Totty W, Sotelo A, Atkinson J (2002). Synovial lipomatosis (lipoma arborescens) affecting multiple joints in a patient with congenital short bowel syndrome. J Rheumatol.

[2] Miller RL, Donaldson EK, Giuffre JL (2024). Synovial lipomatosis with extra-articular extension in the arthritic wrist: an unexpected diagnosis. Ann Plast Surg.

[3] Thavasianantham K, Raagul TS, Ganesh A, Elangovan P, P H, Regunathan PS (2024). Unusual presentation of synovial lipomatosis mimicking as septic arthritis of knee: a case report. Cureus.

[4] AbdullGaffar B, Keloth T, Abdulrahim M (2024). Synovial lipomatosis: histopathologic features in a series of five cases. Int J Surg Pathol.

[5] Miladore N, Childs MA, Sabesan VJ (2015). Synovial lipomatosis: a rare cause of knee pain in an adolescent female. World J Orthop.

[6] Youm YS, Cho SD, Cho HY, Kang TW (2017). Bilateral hypoplasia of the medial and lateral menisci. Knee Surg Relat Res.

[7] Hasegawa S, Horie M, Katagiri H, Tsuji K, Muneta T, Koga H (2018). Bilateral hypoplasia of both medial and lateral menisci partially fused with the cartilage surface of the tibial plateau. Orthopedics.

[8] Utkan A, Ozkan G, Köse CC, Ciliz DS, Albayrak AL (2010). Congenital absence of the medial meniscus associated with lipoma arborescens. Knee.

[9] Khatri A, Mahajan N, Agarwal A, Gupta N (2019). Synovial lipomatosis with chondroblastoma in an 8-year-old female: a previously unreported entity. J Orthop Case Rep.

[10] Soler T, Rodríguez E, Bargiela A, Da Riba M (1998). Lipoma arborescens of the knee: MR characteristics in 13 joints. J Comput Assist Tomogr.

[11] Zhu T, Deng C, Zhou Y (2025). Age dependent morphological changes of the normal meniscus in children based on large scale MRI analysis. Sci Rep.

[12] Abusara Z, Andrews SH, Von Kossel M, Herzog W (2018). Menisci protect chondrocytes from load-induced injury. Sci Rep.

[13] Lee HH, O'Malley MJ, Friel NA, Chu CR (2013). Effects of doxycycline on mesenchymal stem cell chondrogenesis and cartilage repair. Osteoarthritis Cartilage.

[14] Gao KT, Xie E, Chen V (2023). Large-scale analysis of meniscus morphology as risk factor for knee osteoarthritis. Arthritis Rheumatol.

[15] Rohde MS, Trivedi S, Randhawa S (2023). Pediatric meniscus morphology varies with age: a cadaveric study. Knee Surg Sports Traumatol Arthrosc.

[16] Kwon OS, Sun Y, Enobun B (2019). Meniscal repairs in the adolescent knee: can the number of fixation sites improve outcomes?. Orthop J Sports Med.

[17] Kim TK, Phillips M, Bhandari M, Watson J, Malhotra R (2017). What differences in morphologic features of the knee exist among patients of various races? A systematic review. Clin Orthop Relat Res.

[18] Natera L, Gelber PE, Erquicia JI, Monllau JC (2015). Primary lipoma arborescens of the knee may involve the development of early osteoarthritis if prompt synovectomy is not performed. J Orthop Traumatol.

[19] Wang CK, Alfayez S, Marwan Y, Martineau PA, Burman M (2019). Knee arthroscopy for the treatment of lipoma arborescens: a systematic review of the literature. JBJS Rev.

[20] Huang Y, Liu H, Wang Y, Chu X, Liu H, Wang H (2022). Imaging features of lipoma arborescens. Acta Radiol.

